# Cancer Treatment Goes Viral: Using Viral Proteins to Induce Tumour-Specific Cell Death

**DOI:** 10.3390/cancers11121975

**Published:** 2019-12-07

**Authors:** Jasmine Wyatt, Manuel M. Müller, Mahvash Tavassoli

**Affiliations:** 1 Department of Molecular Oncology, King’s College London, Guy’s Hospital Campus, Hodgkin Building, London SE1 1UL, UK; Jasmine.Wyatt@kcl.ac.uk; 2 Department of Chemistry, King’s College London, 7 Trinity Street, London SE1 1DB, UK; Manuel.Muller@kcl.ac.uk

**Keywords:** Apoptin, NS1, E1A, E4orf4, Cancer, Apoptosis

## Abstract

Cell death is a tightly regulated process which can be exploited in cancer treatment to drive the killing of the tumour. Several conventional cancer therapies including chemotherapeutic agents target pathways involved in cell death, yet they often fail due to the lack of selectivity they have for tumour cells over healthy cells. Over the past decade, research has demonstrated the existence of numerous proteins which have an intrinsic tumour-specific toxicity, several of which originate from viruses. These tumour-selective viral proteins, although from distinct backgrounds, have several similar and interesting properties. Though the mechanism(s) of action of these proteins are not fully understood, it is possible that they can manipulate several cell death modes in cancer exemplifying the intricate interplay between these pathways. This review will discuss our current knowledge on the topic and outstanding questions, as well as deliberate the potential for viral proteins to progress into the clinic as successful cancer therapeutics.

## 1. Introduction

Cancer is a major public health problem, of which incidence and mortality is rapidly growing worldwide. An estimated 9.6 million deaths were attributed to cancer in 2018 making it the leading cause of death globally [1]. Mainstream therapeutic approaches for cancer include chemotherapy and irradiation. However, for many types of cancers these treatments are largely ineffective due to the lack of selectivity for tumour cells over normal cells. Therefore, there is a demand for new innovative cancer treatments. The intrinsic capability for some viruses to kill cancer has been acknowledged for more than a century, nonetheless the promising research in this area has not yet been translated into successful clinical use—possibly due to the complex interactions between the virus and host immunity which is not yet fully understood (oncolytic viruses reviewed fully in [2]). Consequently, research has begun to investigate the antitumor activities of some of the individual protein components within these viruses. Several of these have been identified to exhibit tumour-selective toxic capacity to a similar extent as the whole virus, and therefore have gained interest in the field of cancer research [3].

Anti-cancer proteins found encoded within viruses are from a variety of origins including the chicken anaemia virus, rodent parvoviruses and human adenoviruses (Table 1). They are known to promote cell death via numerous modes of action; however, thus far the precise mechanism(s) of tumour-selectivity are largely unknown and the literature around this area is somewhat controversial. Nonetheless, the research does point to the existence of a paradoxical mechanism of action associated with a tightly controlled balance between death sparing and death promoting depending on the cellular context. This review will discuss our current understanding on using viral proteins to induce tumour-selective death, as well as examining the striking similarities between these proteins and present some unresolved questions in the field.

## 2. Viral Pro-Death Anti-Cancer Proteins

### 2.1. Apoptin

Apoptin was the first viral anti-cancer protein to be described, which originates from the chicken anaemia virus (CAV) isolated in Japan in 1979. CAV is a small DNA virus comprising of a single-stranded circular DNA genome and is a member of the *Gyrovirus* genus [10]. CAV infects young chicks, of which the major target is hematopoietic cells in the bone marrow and T-cell precursors of the thymus. Infection of these cells by CAV results in cellular death and consequent anaemia and thrombocytopenia [11]. CAV encodes for 3 proteins, of which VP3 a small 13.6 kDa non-structural protein is responsible for the cytopathogenicity observed and was later termed Apoptin. Interestingly, the toxic action of Apoptin is not only constrained to these cell types and has been found to selectively induce cell death in transformed mammalian cells, whilst sparing healthy cells [12,13].

**Figure 1:**
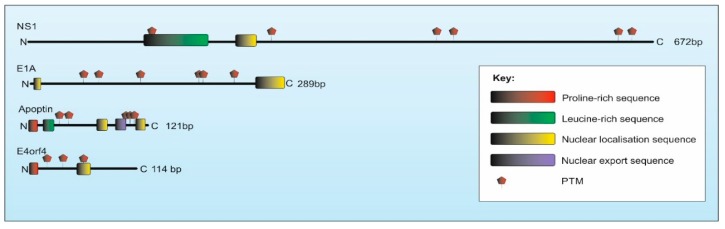
The key domains and sequences of the viral proteins discussed in this review. Post-translational modifications occur in the form of phosphorylation. All are thought to be intrinsically disordered, except for the much larger NS1 which known to have a distinct folded N-terminus (residues 1–255).

The apoptin protein is composed of 121 amino acids and is rich in proline, serine and threonine residues (see Figure 1). At the C-terminus, Apoptin contains regions important for cellular localisation of the protein including; a bipartite nuclear localisation sequence (NLS) located between residues 82-88 (NLS1) and 111-121 (NLS2), as well as a nuclear export signal (NES) between residues 97–105 [14,15]. Apoptin has been reported to exist as an intrinsically disordered protein (IDP), with very little secondary structure. Nonetheless, the active form of the protein forms non-covalent globular multimers, possibly composed of 30–40 monomers. This interaction is thought to be driven through the hydrophobic region at the N-terminus of the protein comprised of a stretch of prolines between residues 8–28 and leucines between amino acids 33–46 [16]. Unfortunately, high resolution structural data of the multimeric apoptin complex is not available currently.

There are various potential post-translational modification (PTM) sites within the Apoptin protein. Studies have shown that in cancer cells Apoptin is frequently phosphorylated, particularly at the site threonine-108, which is believed to be important for the toxic potential of the protein [17]. This PTM occurs by tumour-specific kinases such as an isozyme of protein kinase C (PKCβ) which was found to interact with Apoptin in colorectal cancer cells [18]. Even so, other sites within the protein seem to be important for oncotoxicity including the adjacent threonine residues -106 and -107. Rohn et al. established that mutation of the this key threonine-108 to a non-phosphorylatable residue causes threonine-107 and 106 to become opportunistically phosphorylated and tumour selective death is thereby maintained [19]. Nonetheless, it is still not recognised whether each residue becomes individually phosphorylated in cancer cells, and whether the amount of phosphorylation can directly correlate to the amount of cell death observed. More recently, studies identified two novel checkpoint kinase (Chk) consensus sites (threonine-56 and -61) which can also be phosphorylated in cancer cells and are also vital sites for modification and subsequent cell death [20].

Interestingly, the PTM of Apoptin has been proposed to drive a nuclear accumulation of the protein. The subcellular localisation of the protein is an additional characteristic which appears to correlate to the proteins ability to induce cell death. In normal cells, Apoptin is chiefly found within the cytoplasm, whereas in tumour cells apoptin is localised to the nucleus. Several studies have confirmed that the nucleo-cytoplasmic shuttling of Apoptin is regulated by both the NLS’s and NES’s within its structure. The NLS of Apoptin appears to be active in both cancerous and normal cells, as Apoptin freely translocates in the nucleus of healthy cells, albeit to a lesser degree [21]. Mutation within either NLS abrogated nuclear accumulation of Apoptin in cancer cells, suggesting that both sequences are required for efficient nuclear targeting [14]. The translocation of the protein out of the nucleus is most likely facilitated by the NES which can be recognised by the nuclear export protein CRM1 [14]. Observed phosphorylation of the protein directly adjacent to the NES provoked the theory that PTM by tumour specific kinases masks the NES, blocking its recognition by CRM1 and thus triggering a build-up of the protein in the nucleus of transformed cells. This fascinating theory has been supported by numerous studies, including the creation of a phospho-mimic of Apoptin (Threonine108>Glutamic acid) which begins to accumulate also in the nucleus of healthy cells. With that said, fusion of Apoptin to a strong NLS drives a build-up of the protein in the nucleus of healthy cells but is not sufficient to induce apoptosis [14]. Together, these observations suggest that additional events are required for the activation of Apoptin but does imply that the protein needs to locate to the cell nucleus in order to induce cell death in transformed cells.

In 2011, a human homologue of the CAV named the human gyrovirus (HGV) was identified and is the first human-infecting member of the *gyrovirus* genus recognised so far [5]. HGV is a small (2.3kb) single stranded DNA virus. One recent study demonstrated that the virus is found in numerous humans, especially in several immunocompromised patients. Nevertheless, the pathogenic potential of HGV remains poorly understood [22]. Despite the homology between the CAV and HGV genomes, the overall sequence identity of the Apoptin protein is relatively low (31%). Regardless, the protein has high sequence conservation within some important protein domains including the nuclear localisation and export signals, as well as the leucine rich region and PTM sites. Studies have revealed that this variant of Apoptin has a similar cancer death inducing ability as its CAV counterpart, as well as a similar subcellular localisation and phosphorylation pattern [23]. Given that research on the HGV is relatively recent, more thorough characterisation of the HGV-derived Apoptin protein is needed before the protein could possibly progress clinically.

Torque teno virus (TTV) is a small non-enveloped human virus classified to the *Alphatorquevirus* genus discovered in Japan in 1997 [6,24]. TTV is a ubiquitous virus which is found in more than 90% of the general population and is transmitted in all ways [25]. Despite research over the past 22 years and extremely high prevalence, its involvement in specific diseases is not understood. Some links have been made with several illnesses such as acute respiratory disease, liver disease and cancer, but this needs to be further investigated [26,27,28]. TTV comprises of a single stranded DNA genome of 3.8kb in length and the virus shows a number of genomic similarities to the CAV [29]. TTV, like CAV, encodes for a protein in open reading frame 3, which has been shown to induce apoptosis in the context of the virus. This protein also explicitly causes cell death in transformed cells over healthy cells and has since been termed the TTV apoptosis-inducing protein (TAIP) [30]. Unlike Apoptin, TAIP is localised to the cytoplasm of cancerous cells but has been shown to localise to chromatin in apoptotic cells. However, thus far there is very limited research on the death-inducing ability of this protein. The TAIP protein is composed of 105 amino acids (12 kDa), and has several similar protein domains to the CAV-Apoptin including a conserved hydrophobic region at the N-terminus [31]. Additionally, TAIP has high proline content, as well as potential PTM sites due to the high number of threonine residues. 

### 2.2. NS1

Previous research has shown that some rodent viruses have intrinsic oncosuppressive properties. Amongst the viruses studied for their anticancer qualities the parvovirus H-1 (H-1PV) and the minute virus of mice (MVM) have been the most extensively researched [32,33]. The natural hosts of these viruses are rats and mice respectively and they share many biological features. These viruses belong to the *Parvovirus* genus and are single stranded non-enveloped DNA viruses with genomes of around 5.1kb [24]. Both viruses show no clinical or pathological signs after infection. However, experimental infections of rodent foetuses do show some discrete developmental changes [34]. The parvovirus genome encodes for at least six non-structural proteins, of which the non-structural-1 protein (NS1) exerts a range of functions including cytotoxicity [35]. Interestingly, when the protein is expressed outside of the virus it can kill transformed cells preferentially over healthy cells [36,37].

The encoded NS1 protein shows 91% sequence identity between H-1PV and MVM. Both encode for a 672-amino acid protein (83 kDa) made up of several domains which allow the protein to be highly multifunctional (Figure 1). These functions are known to include endonuclease and helicase activity [38]. Unlike Apoptin, NS1 has a partial X-ray crystal structure, which has demonstrated that the protein has a more distinct structure of alpha helices and beta sheets, making it the only protein discussed in this review with distinctly folded domains [39,40]. NS1 is also known to self-associate, although the exact size of these oligomers is still unclear [41,42]. The NS1 protein contains several regions similar to those described within other viral proteins with cell death-inducing capabilities, including a bipartite NLS between 194–216 [43,44]. It also contains a leucine rich domain located at residues 180–380 which is vital for multimerization and sites of phosphorylation at residues 283, 403, 435, 473, 585 and 588 which are known impact the function of the protein [45,46,47,48,49,50].

Comparable to Apoptin, the PTM of this protein is carried out by tumour specific kinases. Various studies have shown that this phosphorylation is carried out primarily by another isoform of PKC (PKCλ) [46,51,52]. Nucleo-cytoplasmic transport of NS1 also seems important for its toxic function. Similarly to Apoptin, a significant proportion of the protein seems to reside in the nucleus, however it has been noted around 30% of the protein translocates back to the cytoplasm [43]. This shuttling is reliant on the NLS, as mutation of key residues here abolishes the ability of the protein to gain entry into the nucleus of cancer cells [53]. The link between phosphorylation and subcellular localisation and toxicity has been explored and demonstrated that the modification of specific residues is important for the toxicity of the protein (Threonine-435 and Serine-473) as mutation here abolishes cell death. However, none of the phospho-null mutants altered the subcellular localisation of the protein in cancer cells suggesting phosphorylation does not eliminate nuclear shuttling of NS1 [45].

### 2.3. E1A

Adenoviruses (AdVs) are non-enveloped viruses with an icosahedral capsid containing a double stranded linear DNA genome of around 26–45 kb in size [54]. Adenoviruses are found across all vertebrates with species-specificity. Human adenoviruses (HAdVs) are classified in the genus *mastadenovirus* and so far 67 human adenovirus types have been discovered [55]. Clinically, HAdVs can cause a range of diseases including conjunctivitis, gastroenteritis, hepatitis, myocarditis, and pneumonia; of which most occur in children [55]. Early observations that AdVs could kill cervical cancer cells prompted human clinical trials in the 1950s, where tests began in using these viruses to infect and lyse cancer cells in a process termed viral oncolysis [56,57]. After viral infection, the first gene to be expressed is the early region 1A gene (E1A), which has been most extensively characterised in types 2 and 5 of HAdV’s [58]. Interestingly, outside the context of the virus, expression of this protein is enough to suppress tumour growth in in a number of human cancer cell lines, whilst sparing healthy cells [59]. Notably, E1A also has the ability to transform certain cell lines, indicating that this protein has context-specific transforming and anti-oncogenic activity, further demonstrating the delicate balance between life and death in cancer [59].

The gene products of E1A are expressed in two distinctively spliced messenger RNAs (mRNAs), the 13S (289 amino acid) and the 12S (243 amino acid), which encode for a 32 kDa and a 26 kDa protein, respectively [60,61]. The encoded proteins are identical bar the additional 46 amino acid addition in the larger protein and are virtually identical between HAdV2 and 5. To note, the larger protein has been studied most in the context of cancer-specific death. E1A has no known intrinsic enzymatic capabilities and has been predicted to have no secondary structure and probably forms an IDP with a weak propensity to form some alpha helical regions [62]. The protein tends to exist as a monomer, but may associate with itself to form homo-dimers [63]. A deeper look at the primary sequence of E1A shows that the protein contains a high (15%) proline content and a bipartite NLS between residues 258–289 (NLS1) and an additional NLS (NLS2) between 7–20 [64,65]. More recently a novel non-canonical NLS was defined between residues 30–69 [66]. NLS1 is known to interact with the nuclear import protein importin alpha 3 (Qip1) and in cancer cells E1A accumulates in the nucleus, but also in high amounts in the cytoplasm [67,68]. Phosphorylation can occur at numerous serine residues identified as 89, 96, 132, 185, 188 and 219 [69,70]. This modification can occur by kinases constitutively active in cancer cells such as mitogen-activated protein kinase (MAPK) which phosphorylates serine-185 and -188 specifically [69]. Cyclin-dependant kinases (CDK) have also been found to be extremely important, as well as casein kinase II (CKII) which phosphorylates serine-132, a modification vital for its apoptosis-inducing abilities [71,72].

### 2.4. E4orf4

In addition to E1A, the early region 4 (E4) of HAdV’s was also found to be important for apoptosis induction in the context of the virus [73]. E4 encodes several polypeptides that regulate a number of biological functions including transcription, viral mRNA transport, cell cycle, DNA repair and apoptosis. The protein within this region responsible for apoptosis was identified as the open reading frame 4 (E4orf4) [74]. Like E1A, this protein can induce cell death specifically in transformed cell lines, sparing normal cells [75].

The human E4orf4 encodes a 114-amino-acid protein (in the case of Ad2 and Ad5) (14 kDa). The protein differs slightly between Ad2 and Ad5 by one residue (A67 in Ad2, S67 in Ad5), however this has not been linked to any structural or functional differences and they possess a similar ability to kill cancer cells [76]. The protein has a basic NLS between amino acids 66-75 and is rich in prolines at the N-termini [77]. The protein is known to become phosphorylated on a number of tyrosine residues including 26,42 and 59 which can occur by Src family kinases, a family that is upregulated in numerous tumour lines [78,79]. As with the other viral proteins discussed in this review, the phosphorylation of this protein correlates with its cellular localisation. In this case of E4orf4, the protein locates within nuclear regions of the cell where it begins to accumulate. At the same time however, the protein also begins to rapidly collect in cytoplasmic membrane regions where it is found in membrane blebs [80,81] Mutation of the key tyrosine residues to alanine changes the subcellular localisation of the protein and significantly impairs the killing activity [78]. Although there is no solved structure of this protein, computational modelling has predicted that the protein is highly unstructured, likely existing as an IDP with some alpha helical domains [78].

## 3. Cancer Cell Death Induction Mechanisms

**Figure 2:**
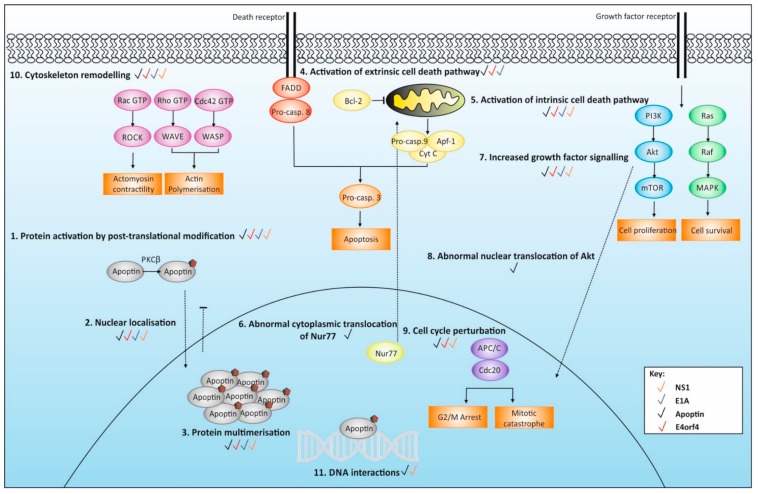
Schematic showing the numerous cellular events which are altered by viral proteins in transformed cells (see key bottom right corner). All tumour-selective viral proteins undergo PTM’s in the form of phosphorylation to become active. This occurs by kinases which may be upregulated in cancer, however the exact kinases may vary between cell-type. Proteins can freely translocate between the nucleus and the cytoplasm via the nuclear localisation and export sequences they all contain. PTM may impair this nuclear transport (evidence shown at least for Apoptin which builds up in the nucleus of cancer cells). Viral proteins can multimerise although it is unclear whether this happens in specific cellular compartments. The figure uses apoptin as an example, which tends to multimerise in the nucleus where it accumulates in aggregates. Components of the parallel apoptosis pathways (intrinsic and extrinsic) can be activated by most of the viral proteins discussed. Collectively though, they all result in some form of caspase activation which typically results in downstream activation of convergent pathways leading to apoptosis. Uniquely, Apoptin can promote the abnormal translocation of Nur77 from the nucleus to the mitochondria, leading to MOMP and thus activation of the intrinsic cell death pathway. Non-canonical modes of cell death may also appear in different forms, which can be altered by viral proteins and will naturally feed into the classical apoptosis pathways further downstream. This includes perturbations of the cell cycle, which could occur when some viral proteins interact with the APC/C. These proteins can all also promote cytoskeleton remodelling through interactions with different family members of Rho GTPases, as well as disrupting/activating growth factor signalling pathways. Overall, the landscape of tumour-selective cell death induced by these viral proteins is extremely complex and probably does not occur through a signal pathway, instead occurring through the activation and crosstalk of multiple intricate pathways.

The mode of action of these viral proteins in cancer-specific induction of cell death is largely unknown and controversial. Granted, several mechanisms have been proposed. One of the major tumour-selective characteristics observed, as discussed in Section 2, is phosphorylation (see Table 2). This is thought to result in a differential cellular localisation within the transformed cell lines, although some proteins show a dissimilar extent of this. For example, Apoptin is seen predominantly in the nucleus of cancer cells, whereas E4orf4 accumulates firstly in the nucleus but later begins to translocate in the cytoplasm. Together, these observed traits are thought to drive cancer cell death, but how exactly does this occur? Interestingly, many of the proposed cell death modes show striking similarities between all the discussed proteins, while the extent and type of cell death depends highly on the host cell environment (similarities summarised in Table 3). However, tumour-selective cell death does not seem to be driven by a single pathway, and possibly occurs as the result of the activation and interplay of several pathways (see Figure 2).

### 3.1. Activation of Classical Cell Death Pathways

Regulated cell death is an important physiological process involved in development and tissue turnover, and is often referred to as programmed cell death [82]. This is in direct contrast with accidental cell death, caused by overwhelming cellular damage. Cell death can be characterised by distinct morphological changes which has led to the classification of three different forms. Type 1 cell death or apoptosis, which exhibits distinct morphological changes in the cell including cell shrinkage, membrane blebbing, chromatin condensation and fragmentation of DNA. In contrast, autophagy or type II cell death appears as cytoplasmic vacuolization, shadowed by phagocytic uptake and consequent lysosomal degradation. Lastly, type III cell death or necrosis displays no distinctive features of type I or II cell death, but terminates with the rupture of the cellular membrane and release of the cell contents [83]. This classical death pathway classification is still used to date; however, it is important to note that novel signalling pathways that orchestrate cellular death are still being discovered. This includes forms of death such as ferroptosis, a type of death initiated by oxidative perturbations or necroptosis and pyroptosis, both of which are inflammatory modes of cell death (most recent classification of cell death reviewed fully in [84]). So far, these alternate cell death modalities have not been reported to have a role in viral protein oncotoxicity, but it should be noted that there is a high degree of interconnectivity between all the pathways described thus far. 

There are two major pathways that can trigger apoptotic death in mammalian systems: the intrinsic (mitochondrial) and the extrinsic (death receptor), both of which are accompanied by the stimulation of a caspase cascade. The former pathway largely relies on the action of Bcl-2 family members, which can stimulate mitochondrial permeabilization and cytochrome c release [85,86]. Once released to the cytosol, cytochrome c induces the assembly of the apoptosome which leads to subsequent caspase activation [87]. On the other hand, the extrinsic cell death pathway is triggered by perturbations in the extracellular environment and is directly stimulated by death factors such as Fas or TRAIL. These can oligomerise, allowing for the recruitment of FADD and caspase 8 [88,89]. Pathways in either types of cell death converge in the same downstream pathway via activation of the caspases, ultimately resulting in the degradation of cytoskeletal and nuclear proteins, DNA fragmentation and the formation of apoptotic bodies. 

#### 3.1.1. Importance of Caspases

One common feature in viral protein induced toxicity is the activation of caspases which is a common event of both the intrinsic and extrinsic cell death pathways. This includes both the stimulation of upstream initiator caspases (-8 and -9) and the downstream executioner caspases (-3, -6, and -7) [90,91]. Several studies have utilised broad spectrum caspase inhibitors to demonstrate the importance of these molecules, as inhibition of caspases is protective against CAV-Apoptin, HGV-Apoptin, NS1, and E1A induced cancer cell death [92,93,94,95]. Interestingly, studies have shown that this phenomenon in E4orf4 is not as straight forward and is most likely cell-line specific, as inhibition of caspases does not fully protect against cell death in ovarian cancer cells but does inhibit death of lung carcinoma lines [96,97].

More specifically, the executioner caspase 3 has been shown to be collectively important for cancer cell death induction by viral proteins. Caspase 3 activation by Apoptin from both CAV and HGV has also been shown in a huge variety of cancer cell lines [92,93,98,99,100]. NS1 and E1A expression is also sufficient to induce activation of caspase 3 [94,101]. Notably, caspase 3 is activated during the overexpression of E4orf4 in cancer cells but doesn’t seem to be vital for the induction of cell death [97]. Other caspases 9 and 7 are also significant for CAV-Apoptin and NS1 induced cell death [92,93,94,102]. 

#### 3.1.2. Mitochondrial Cell Death

The initiation of mitochondrial cell death by means of mitochondrial outer membrane permeabilization (MOMP) is a universal effect of these viral proteins. The pro-apoptotic proteins targeting the mitochondria Bax and Bak are important in Apoptin cancer toxicity as treatment with Apoptin increased the expression of Bax and cells devoid of both are strongly protected against cell death [92,102]. Moreover, studies have presented that Apoptin treatment leads to a loss of mitochondria membrane potential and subsequent release of cytochrome c [92,102,103]. Similarly, the highly related HGV-Apoptin protein also relies on the activity of Bax and Bak, as jurkat cells that lack expression are protected from apoptosis. Moreover, treatment with this protein has been shown to induce MOMP and consequent release of cytochrome c [93]. The activity of E4orf4 poses a similar mechanism, as treatment increases the expression of Bax [97]. Alternatively, there have been conflicting studies on the role of the pro-survival protein Bcl-2 which acts to inhibit Bax and Bak. In the case of CAV-Apoptin contradictory results have described that Bcl-2 accelerates apoptin induced cell death, whilst others have shown it inhibits toxicity [92,99,104,105]. Conversely, E4orf induced cell death is not inhibited by Bcl-2, but the activity of E1A cell death is blocked by Bcl-2 [80,106].

Another interesting aspect of this pathway is the role of Nur77, a transcription factor implicated in apoptosis. This transcription factor is known to induce apoptosis by upregulating a number of pro-apoptotic genes including FasL and TRAIL [107,108]. Nur77 also has a less well-known function which is to promote MOMP [109]. In normal conditions Nur77 is found in the nucleus but regulated by phosphorylation, which occurs by MAPK and AKT. Phosphorylation of Nur77 drives the abnormal translocation of the transcription factor to the mitochondria where it causes MOMP ensuing cytochrome c release [110]. The activity of this transcription factor in the context of viral protein cancer toxicity has been mostly studied in CAV-Apoptin. When apoptin is expressed, Nur77 can be seen to translocate to the cytoplasm where it does indeed act to causes cytochrome c release and subsequent apoptosis. Additionally, downregulation of Nur77 protects cancer cells against apoptin-induced cell death [90,103]. Interestingly, Nur77 is known to interact with Bcl-2 and turn it from anti- to pro-apoptotic which may explain the conflicting results for this protein [111]. Nur77 is also important for HGV-Apoptin as treatment triggers cytoplasmic translocation of Nur77 93 Nevertheless, although less is known about this transcription factor in other viral protein mechanisms, studies have found that Nur77 interacts with an E1A binding partner p300, and the overexpression of E1A can repress Nur77 transactivation [110,112]. 

#### 3.1.3. Death Receptor-Mediated Toxicity

Although treatment of cancer cells with these viral proteins is frequently considered to induce mitochondrial cell-death as described above, there is some limited evidence to suggest possible activation of extrinsic cell death pathways. This further reinforces the idea that the cell death mechanisms used by these proteins is context dependant. For example, Guelen et al. showed that CAV-Apoptin co-localises with overexpressed FADD in the cytoplasm [113]. Further to this, treatment with apoptin led to the expression of caspase 8, a caspase exclusive to the death receptor cell death pathway [102]. On the contrary, some studies have shown that cells are still sensitive to both CAV and HGV apoptin death after blocking FADD [90,93]. HAdV proteins may act on the extrinsic pathway in a similar way to apoptin. Dominant negative mutants of caspase-8 and the death receptor adapter protein FADD/MORT1 was shown to inhibit E4orf4-induced apoptosis in cancer cells, suggesting that E4orf4 requires this pathway [97]. Similarly, E1A treatment promotes death receptor mediated activation of caspase-8 [114].

#### 3.1.4. P53-Independence

p53 is a nuclear transcription factor and transactivates numerous target genes involved in the induction of cell cycle arrest and/or apoptosis [115]. Importantly, all viral proteins discussed in this review act independently of p53 [13,74,93,96,116,117]. p53 has been discovered to be mutated in more than 50% of human malignancies and thus the fact that these proteins can act independently of p53 is an extremely important characteristic required for the future development of these proteins as cancer therapeutics [118].

### 3.2. Non-Canonical Modes of Cell Death

Many of these proteins have been shown to induce a non-canonical mode of cell death inconsistent with typical apoptosis. Nonetheless, the activation of these pathways likely relies on the interplay and subsequent activation of classical cell death pathways (as discussed in Section 3.1) and seem to be extremely context dependant, likely varying based on the physiological state of the cell. 

#### 3.2.1. Perturbations of The Cell Cycle

It has long been recognised that there is a crosstalk between the cell cycle and apoptosis, as both processes are regulated by a similar set of genes [119]. Manipulation of the cell cycle may induce or prevent apoptosis depending on the cellular context, but typically extended mitotic arrest ultimately results in cellular death [120,121]. Some of the viral proteins have been shown to induce cell cycle arrest at the G2/M phase including CAV and HGV-Apoptin, E4orf4 and NS1 in cancer cells [122,123,124,125]. Likewise, cells treated with apoptin display clear abnormal mitotic figures, which may explain the pause in this stage of the cell cycle [126]. One major control of cellular division is the anaphase promoting complex or cyclosome (APC/C), which is a multisubunit E3 ubiquitin controlling cycle progression through the degradation of vital regulators. Specifically, the complex regulates advancement through the mitotic phase and controls entry into the S phase [127]. One interesting theory for the mechanism of action of these viral proteins is the existence of a direct interaction with the APC/C and thus disturbing cell cycle progression and ultimately driving apoptosis. E4orf4 and Apoptin from both CAV and HGV has been shown to interact directly with subunits of the APC/C [122,123,124]. Although APC/C has not been studied in the context of E1A cancer cell toxicity, it has been shown that E1A interacts with APC components such as CBP/P300 in the context of viral transformation and likely has similar effects in tumour cells, so this link needs to be further explored [128,129].

#### 3.2.2. Cytoskeleton Remodelling

The execution phase of apoptosis is signified by dramatic morphological changes including cell shrinkage and plasma membrane blebbing [83]. To accomplish this, dying cells undergo defined cytoskeleton reorganizations and caspase-mediated digestion of cytoskeletal proteins [130]. As well as being important for the execution phase of apoptosis, alterations in cellular architecture have been implicated as a direct regulator of reactive oxygen species (ROS) release from the mitochondria and thus can also drive cell death via the intrinsic cell death pathway [131].

Expression of viral proteins has been shown to result in distinct alterations of the cancer cell architecture. Expression of CAV Apoptin was shown to initially localise to the cytoplasm in a filamentous pattern resembling actin stress fibres at early stages of expression only in cancer cell lines. Nonetheless, this same study noted that these filaments were able to form without an intact actin stress fibre network when treated with cytochalasin D [113]. This therefore needs to be researched further. However, Apoptin coimmunoprecipitates with alpha-tubulin, beta-tubulin and beta-actin, suggesting that the protein does positively associate with filamentous networks nevertheless [122]. The cytoplasmic translocation of E4orf4 has been shown to be associated with changes in the filamentous actin cytoskeleton and stably accumulated to perinuclear vesicles and cortical sites forming membrane protrusions [132]. E1A proteins also disrupt actin stress fibres in the context of viral infection in transformed rat cells [133]. Similarly, NS1 notably causes cytoskeleton collapse which manifests in degradation of filamentous actin and vimentin structures [134].

Rho family small GTPases such as RhoA, Cdc42, and Rac can control the actin cytoskeleton dynamics. Typically, Rho proteins regulate the formation of actin stress fibre formation, whereas Cdc42 and Rac stimulate actin polymerisation via the Arp2/3 complex [135]. There is evidence to suggest that E4orf4 killing relies on the activation of several of the Rho GTPase family members. These each make distinct contributions to the actin dynamics in the cell, resulting in subsequent abnormal perinuclear actomyosin structures which have been suggested to perturb vesical traffic and organelle membrane dynamics triggering cell death. Further to this, inhibition of actin dynamics dramatically impairs E4orf4 killing [132]. The E1A protein can interact with the Rac-Cdc42 pathway in transformed rodent cells. This can lead to the reorganisation of the actin cytoskeleton with increased filopodial and lamellipodial production and subsequent enhanced cellular motility and a loss of contact inhibition, which perhaps drives oncotoxicity [136]. Differently, the cytoskeleton re-arrangement potential of NS1 in cancer cell lines seems reliant on an interaction with the catalytic subunit of casein kinase II [137]. This is thought to disrupt the cytoskeleton through the activation of gelsolin which severs actin filaments and also through the suppression of N-WASP which usually promotes actin polymerisation [134,138]. This specific type of alteration may possibly impact cancer cells more greatly since they already lack a rigid cytoskeleton.

#### 3.2.3. Hijack of Growth Factor Signalling Pathways

The phosphatidylinositol-3-kinase (PI3K)/serine/threonine kinase signalling pathway has a well-established role in cell survival and is important in the regulation of the cell cycle also. In numerous cancers, the constitutive activation of this pathway is known to drive malignant progression [139]. In this pathway, AKT, a serine-threonine kinase, is activated at the plasma membrane after recruitment by phosphatidylinositol 3,4,5-phosphate generated by the PI3-K. AKT has a number of functions, classically thought to moderate substrates involved in cell proliferation. More recently, components of the PI3K pathway have been demonstrated to translocate to the nucleus where they exert largely undefined functions [140]. Although the PI3K pathway is generally acknowledged as a cell survival pathway, emerging evidence suggests that it may have a dual role in cell survival and cell death and may contribute to cell death pathways including autophagy depending on the stimuli [141,142,143].

Fascinating reports have shown that Apoptin expression in cancer cells leads to hyperactivation of these pathways. Maddika et al. demonstrated that Apoptin interacts with the p85 regulatory subunit of PI3-K in several cell lines leading to its constitutive activation. Furthermore, the inhibition of this pathway severely impairs apoptin induced cell death and retains the protein in the cytoplasm of transformed cells [144,145]. This same research group also showed that during apoptin-induced cell death AKT becomes activated and translocated to the nucleus, which results in the subsequent activation of CDK2 [146]. Interestingly, CDK2 has been previously suggested to be the principle kinase that phosphorylates apoptin and is vital for apoptin-induced cancer cell death. Likewise, E1A has been found to interact with insulin receptor substrate (IRS) components which in turn leads to constitutive activation of the PI3-K pathway [147].

Viral proteins from HAdVs demonstrate different interactions with growth factor signalling pathways. E1A was shown to supress epidermal growth factor receptor (EGFR) expression leading to decreased activation of growth factor pathways in head and neck cancer cells. In this same study, exogenous expression of EGFR can protect cancer cells from E1A inducted apoptosis [148]. Similarly, E1A expression represses AKT phosphorylation and activation which leads to decreased growth factor signalling in breast cancer cell lines [149]. Likewise, E4orf4 acts in similar way, as a short peptide sequence of the protein blocks the PI3-K pathway and subsequent signalling [150]. In agreement with this, E4orf4 expression counteracts the constitutive activation of the PI3K pathway when activated with Ras mutation [151]. Contrasting studies have demonstrated a different interaction between the adenoviral proteins and the PI3K pathway. For example, it has been shown that E1A can activate the PI3K pathway in quiescent primary small airway epithelial cells. In this same cell line, E4orf4 induces phosphorylation of p70 downstream of this same pathway and stimulates mTOR [152]. The parvovirus NS1 harbours a potential AKT phosphorylation site at residue T278 that is also conserved across the viruses this protein originates from, but this link has not been explored as of yet [39].

#### 3.2.4. DNA Interactions

Direct DNA binding and/or interactions is an important characteristic some of the discussed viral proteins. For example, the N- or C-terminus of the Apoptin protein can bind to double stranded DNA (dsDNA). Specifically, Apoptin binds to dsDNA to form large nucleoprotein complexes, by binding to at least two dsDNA docecamers per multimer [153,154]. This interaction occurs in a sequence non-specific manner; however the protein may have a preference for fibre ends as the protein has an increased affinity for small (<250bp) fragments of DNA. In addition to this, electron microscopy studies have shown that apoptin is found largely in heterochromatin areas in cancer cells, indicating that apoptin could have a role in regulating gene expression [154]. However, apoptin does not require de novo macromolecular synthesis for toxicity and so apoptin is not thought to act as a transcriptional regulator [14]. Nonetheless, it could be postulated that apoptin interactions with DNA result in aberrant protein-DNA structures which could possibly induce cell death, this link needs to be further explored. Similar to this, the HAdV protein E4orf4 is known to associate with chromatin remodelling factors such as Acf1 in mammalian cells which subsequently recruits the protein to chromatin [155]. Although it is unknown what the effect of this interaction is, it could be predicted to alter local chromatin structure. Although there is until now no evidence to suggest that NS1 interacts directly with DNA, NS1 treatment of cancer cells leads to specific nicks in DNA at sequences which occur frequently in mammalian genomes. This has been speculated to occur due to the intrinsic nuclease activity of the protein and thus the precise interaction between the protein and DNA still needs to be determined [156].

## 4. Investigating the Therapeutic Potential of Viral Proteins in Cancer

To explore the therapeutic potential of these proteins in cancerous cells numerous delivery methods have been explored (see Table 4). Non-viral gene delivery systems are increasingly popular due to advantages in safety, stability, ease of production and lower costs as compared to viral systems [157]. Non-viral pre-clinical studies with viral proteins have so far included the direct delivery of the pro-death gene product within plasmid DNA. For example, direct injection of apoptin-coding plasmids into mice lung carcinomas was performed in conjunction with interleukin-18, which lead to successful delivery of the protein and resulting tumour regression in this model [158]. Similarly, both apoptin and E4orf4 coding DNA has been delivered to murine melanoma models using electroporation. This method lead to tumour growth inhibition during the treatment, but cessation of therapy caused tumour re-growth suggesting gene delivery via this method is not effective [159].

More innovative ways of gene delivery have also been used. For instance, human serum albumin (HSA) which is known to accumulate in tumours, was fused to the apoptin gene to form the resulting plasmid HSA-PEI-pcDNA-Apoptin. This plasmid was shown to be efficiently taken up into the breast cancer cell line MCF7 and also supress tumour growth in nude mice models in vivo [160]. Recently, a novel polyamidoamine dendrimer (PAMAM) was used as a vehicle for apoptin gene delivery to glioma cell lines. These complexes were shown to induce intracellular internalization by endocytosis, and are released from the lysosome resulting in toxicity to glioma cell-lines [161]. Lipid complexes are also useful to deliver DNA. Studies using a complex of the E1A gene with 3β[*N*-(*n*′,*n*′-dimethylaminoethane)-carbamoyl] cholesterol/dioleoylphosphatidyl-ethanolamine (tgDCC-E1A) for gene delivery have encouraging results, as liposome mediated E1A gene transfer significantly inhibited growth of ovarian cancers in treated mice [162]. This idea has since moved into phase I study to determine the maximal tolerated dose and maximum biologically active dose of tgDCC-E1A for the treatment of breast and/or ovarian cancers [163].

Viral gene delivery has advantages over non-viral systems as they are much more efficient at gene transduction in most cell-types. These systems also come with problems however, such as immunogenicity with serious side effects, possibility of insertional mutagenesis and a limit in the size of DNA delivery [157]. Viral-mediated delivery however seems the most promising delivery method of these proteins and several studies have demonstrated this both in vitro and in vivo. Replicative-deficient HAdV’s with mutated E1 regions have been used previously for the treatment of cancer. Instead, the E1 regions can be replaced with the therapeutic gene of choice under the control of an exogenous promotor. For example, the E1A region was replaced with CAV apoptin gene and this method of delivery has been demonstrated to be very successful in a range of cancer cell lines and mouse models [100,164,165,166,167] Conditionally replicative adenoviral vectors have also been utilised. CAV-Apoptin was delivered in this way to gastric and hepatocarcinoma cells [168,169,170]. 

It is not surprising that both E1A and E4orf4 have been delivered within the viruses they originated from. Replication deficient HAdV vectors containing the E1A gene are efficient for the delivery of the protein product and subsequent toxicity within ovarian clear cell carcinoma cells [171]. Replicative AdV’s have also been used for transfer of these proteins to cancer and this has been demonstrated in one study which expressed both the E1A and E4 gene under the control of tissue specific promoter is good for the treatment of prostate cancer [172]. More recently a dual replicative AdV (Ad-Htert-E1A-Apoptin) expressing both Apoptin and E1A was to be extremely effective in treating lung metastasis in a mice model [173,174]. Lenti-viral mediated delivery has also been used for the delivery of CAV-Apoptin to both cellular models and mice models [175,176]. Moreover, Baculoviral delivery has been shown to work well for the delivery of Apoptin both in vitro and in vivo [177].

Several features of naturally occurring adenoviruses can be adjusted to complement tumour-specific replication and toxicity. A good example of this is the development of the HAdV ONYX-015 which contains wild type E1A and E4, but contains an 827bp deletion in the E1B gene. This modified virus produced encouraging results in the early phases of clinical trials and has since progressed to a clinical phase III study combined with standard cisplatin chemotherapy for the treatment of head and neck squamous cell carcinoma (HNSCC) [178]. Another similar example is the development of the HAdV Ad5/3-D24-GMCSF, also termed ONCOS-102. ONCOS-102 is a chimeric HAdV5/3 with a 24bp deletion in E1A and additionally codes for the granulocyte–macrophage colony–stimulating factor (GMCSF) which is a known inducer of anti-cancer immunity [179]. This has recently moved into a phase I clinical trial for the treatment of late stage solid cancer types [180]. Moreover, using whole rodent parvoviruses for the treatment of cancer also seems promising as these viruses demonstrate an intrinsic oncotoxicity due to the expression of the NS1 protein and also a very low pathogenicity towards humans [181]. In 2011, a phase I clinical trial was launched to study the use of PV’s for the treatment of recurrent glioblastoma multiforme and in 2017 for patients with metastatic inoperable pancreatic cancers [182,183].

Lastly, some studies have tried to deliver the purified viral protein directly to cells by fusing the protein to protein transduction domains (PTDs) or cell penetrating peptides (CPPs). This includes the HIV derived TAT peptide which has shown to allow efficient and rapid uptake of cells in vitro and in vivo. This method of delivery has been used for delivery of apoptin as a secretable TAT fusion protein, as well as E1A [184,185]. Apoptin has also been fused to a PTD4 peptide which is effectively taken up in cell models [186,187]. Likewise, the fusion of Apoptin to a novel CPP named MT23 was demonstrated to have specificity to melanoma cells in a mouse model [188]. Apoptin has also been attached to hPP10 from the C-terminal fragment of the KDM4A which was shown to be good for delivery both in vitro and in vivo [189]. E4orf4 protein has been linked to a cell penetrating peptide called DPT-sh1 and has also been fused to the epidermal growth factor receptor (EGFR), a receptor which is overexpressed in many cancers was shown to be internalised in cancer cell lines and could also inhibit tumour growth in nude mice [190,191].

## 5. Conclusive Remarks

Viral proteins which can induce cancer specific cell death are a promising and innovative therapeutic option for the treatment of numerous cancer types. The surprising number of similarities between viral proteins with this ability from different viral sources have been evaluated within this review, and their potential mechanisms of action have been summarised. So far, a number of pre-clinical and clinical trials have been carried out but there are still several important questions related to their clinical application that need to be explored (see Box 1). These are questions which are vital in extending our knowledge of these proteins and may pave the way for cancer treatment going viral in the future!

Box 1 **Some of the many questions that are still outstanding in the field of anti-cancer viral proteins.**

Outstanding questions:

- Which protein domains are required for selective anti-cancer toxicity or do several of these need to co-operate to achieve this effect?
- Is the intrinsically disordered nature of these proteins essential for their function?
- Is the multimeric characteristic vital for their function? Does this multimerization occur in the nucleus of transformed cells?
- Do the frequently observed PTMs of viral proteins drive cancer-selective toxicity? Are additional, currently unknown PTMs involved?
- Do all the proteins discussed exploit the same mechanism of action? Are these mechanisms cell-type specific?
- Which is the most effective and safest way of delivering cancer-selective toxic proteins to tumours?
- If only single protein domains are absolutely required for cancer toxicity, would it be possibly to develop artificial mini-proteins or peptides which mimic the cancer selectivity of the viral proteins?
- Can cancer-selective viral proteins be used in combination with already FDA approved therapies?

## Figures and Tables

**Table 1:** Table detailing the known viral oncotoxic proteins, as well as the year this virus was isolated and the current therapeutic status.

Protein	Viral Origin	Year Isolated	Therapeutic Status
Apoptin	Chicken Anaemia Virus	1979 [4]	Preclinical stage
	Human Gyrovirus	2011 [5]	Preclinical stage
Taip	Torque Teno Virus	1997 [6]	Little preclinical evidence
Ns1	Parvovirus H1	1960 [7]	Clinical trial phase II
	Mice Minute Virus	1966 [8]	Preclinical stage
E4orf4	Adenovirus	1953 [9]	Clinical trial phase III (in combination)
E1a	Adenovirus		

**Table 2:** Summary of the known post-translation modifications (phosphorylation) that occur on viral proteins once expressed in transformed cells. * Isoform not yet confirmed.

Protein	Modification	Enzyme	Ref
CAV-Apoptin	T 106, 107 and 108	PKCβ1	[18]
	T 56	Cdk1/2	[20]
NS1	T435, S473	PKCλ	[46]
	S283, T403, T585 S588	PKC *	[45]
E1A	S89, S96, S132 and S219	Unknown	[69]
	S185 and S188	MAPK	[69]
	S132	CkII	[72]
E4orf4	Y26, Y42 and Y59	Src kinases	[78]

**Table 3:** Comparison of the main characteristics that may contribute to tumour-selective cell death mediated by the viral proteins discussed in this review.

Characteristics	Apoptin	Ns1	E4orf4	E1a
Intrinsically disordered structure	Yes	No	Yes	Yes
PTM’s in the form of phosphorylation	Yes	Yes	Yes	Yes
Nuclear localisation	Yes	Yes	Yes	Yes
Multimerization	Yes	Yes	Unknown	Yes
P53-Independent Death	Yes	Yes	Yes	Yes
Activation of Caspases	Yes	Yes	Yes	Yes
Cytoskeleton Re-Arrangement	Yes	Yes	Yes	Yes
Alterations in PI3K pathway	Yes	Unknown	Yes	Yes
DNA interactions	Yes	Yes	Unknown	Unknown

**Table 4:** Detailing the (pre-)clinical delivery methods used to deliver viral proteins to cancer.

Method		Apoptin	E4orf4	E1A	NS1
**Viral-Delivery of DNA**	Adenovirus	Yes	Yes	Yes	Yes
	Lentivirus	Yes	/	/	/
	Baculovirus	Yes	/	/	/
**Non-Viral Delivery of DNA**	Direct injection	Yes	/	/	/
	Electroporation	Yes	Yes	/	/
	Polymer coupled	Yes	/	/	/
	Liposomal	/	/	Yes	/
**Protein Therapy**	CPP/PTD’s	Yes	Yes	Yes	/
